# Neuroimaging Study Investigating the Supraspinal Control of Lower Urinary Tract Function in Man With Orthotopic Ileal Neobladder

**DOI:** 10.3389/fsurg.2021.751236

**Published:** 2021-12-07

**Authors:** Wanhua Wu, Yun Su, Hao Huang, Meiwei Chen, Fan Fan, Dingjun Zhu, Kaiwen Li, Zhenghui Guo, Zhiying Liang, Hai Huang

**Affiliations:** ^1^Department of Urology, The Sun Yat-sen Memorial Hospital, Sun Yat-sen University, Guangzhou, China; ^2^Guangdong Provincial Key Laboratory of Malignant Tumor Epigenetics and Gene Regulation, Sun Yat-sen Memorial Hospital, Sun Yat-sen University, Guangzhou, China; ^3^Department of Radiology, The Sun Yat-sen Memorial Hospital, Sun Yat-sen University, Guangzhou, China

**Keywords:** bladder cancer (BC), continence, fMRI, neobladder sensation, orthotopic ileal neobladder

## Abstract

**Introduction:** Recent studies employing functional imaging methodology have revealed reference brain regions of urinary tract function, namely, the midbrain periaqueductal gray matter, thalamus, and cingulate and prefrontal cortices. The orthotopic ileal neobladder is a desirable method for urinary diversion after radical cystectomy, but its supraspinal control remains unknown. We aimed to evaluate brain activity while maintaining urinary urgency and voluntary urinary control in male subjects with ileal orthotopic neobladders by performing functional MRI (fMRI) during a block design experiment.

**Materials and Methods:** Patients were recruited at the Sun Yat-sen Memorial Hospital of the Sun Yat-sen University from October 2017 to May 2019. Two tasks were performed during fMRI scanning: (1) repeated infusion and withdrawal of sterile saline solution into and out of the neobladder to simulate urgency; and (2) repeated contraction of the pelvic floor muscle with a full neobladder to induce inhibition of micturition since the subjects were asked not to urinate. The obtained data were visualized and statistically analyzed.

**Results:** Sixteen subjects were recruited in the study, and data were obtained from 10 subjects: mean age 60.1 years, average postoperative time 20.2 months, and daytime continence rate 100%. The parahippocampus, frontal lobe, vermis, and anterior cingulate cortex were activated with large bladder volumes, and the thalamus and caudate nucleus were deactivated during voluntary urinary control.

**Conclusion:** A complex supraspinal program is involved during ileal orthotopic neobladder control, which is significantly different from that with normal bladders, in which the original intestine visceral volume sensation is preserved.

## Introduction

Bladder cancer (BCa) is the 11th most common cancer among humans and the seventh most common among men worldwide, and its morbidity is increasing, placing a heavy burden on society (1). Radical cystectomy (RC) is the standard treatment for patients with resectable muscular invasive BCa, and orthotopic ileal neobladder (OIN) is becoming the best urinary diversion option after RC. This transition has occurred because OIN provides patients with a favorable cosmetic outcome and better quality of life after surgery (2). With adequate postoperative training, the OIN can share many similar functions with the original bladder, namely, storage of urine, voiding, and continence, allowing patients to return to a close to, if not normal, urinary routine (3). However, lower urinary tract dysfunction remains one of the major concerns following OIN surgery. The daytime and nighttime urinary incontinence rates of OIN are 8–10 and 20–30%, respectively, and the urinary retention rate ranges from 4 to 12% (2, 4). It is important to reduce the incidence of urinary dysfunction of OIN and improve the postoperative quality of life. Good urinary function depends on complex spinal and cerebral neural control. Determining the neural control of the lower urinary tract in OIN is crucial.

In recent years, functional imaging studies, such as functional MRI (fMRI), have succeeded in discovering the brain regions of micturition circuits in physiological and disease conditions, such as urinary incontinence, urinary retention, and overactive bladder. The midbrain periaqueductal gray matter (PAG) and the pontine micturition center (PMC) are the central structures and the modulating supratentorial regions. The PAG is the location of the switch from storage to voiding, and it interconnects with many parts of the forebrain, namely, the basal ganglia, hypothalamus, thalamus, frontal cortex, and limbic system (5–7). In disease situations, micturition-related brain activity changes in active regions and with active intensity. This study aimed to evaluate brain activity while maintaining urgency and voluntary urinary control in male subjects with OIN by performing fMRI during a block design experiment.

## Materials and Methods

### Subjects

Male patients with BCa who underwent laparoscopic RC followed by OIN at the Sun Yat-sen Memorial Hospital were recruited according to the inclusion and exclusion criteria from September 2017 to March 2019.

The inclusion criteria were: (1) more than 3 months after surgery; (2) male sex; (3) right-handedness; (4) no contraindications associated with MRI examination; and (5) a signed informed consent form. The exclusion criteria were: (1) open surgery; (2) history of radiotherapy; (3) history of prostate cancer; (4) abnormal lower urinary tract or diseases that affect abdominal pressure; (5) abnormal craniocerebral anatomy or history of craniocerebral surgery, craniocerebral tumors, or craniocerebral radiation therapy; (6) history of mental illness, Parkinson's disease, Alzheimer's disease, epilepsy, cerebral infarction, or cerebral hemorrhage; (7) claustrophobia or metal implants not suitable for MR examination; (8) other diseases that affect lower urinary tract function (such as spinal cord injury and diabetic peripheral neuropathy); and (9) **urethral strictures** (8, 9).

### Measurements During Scanning

Regarding subject preparation, rechecking for contraindications, and patient education, subjects voided before entering the scanner and were catheterized with 14F double-lumen latex catheters (Weili Medical Devices Co., Ltd., Guangzhou, China). The neobladder was drained of any residual urine. The subjects then lay supine in the scanner (Achieva; Philips Medical Systems, Best, the Netherlands, 3-T magnet) on an absorbent pad. The catheters were connected to an MR injection system (Spectris Solaris EP, MEDRAD, Warrendale, PA, USA).

#### T1-Weighted Imaging

Data were acquired on a Philips 3-T full-body scanner (Achieva; Philips Medical Systems, Best, the Netherlands) equipped with a standard eight-channel-phased array head coil (SENSE XL-Torso coil, Achieva; Philips Medical Systems, Best, the Netherlands). Head movements were restrained with foam padding. Headphones and earplugs were used to reduce the interference of noise.

**Figure 1:**
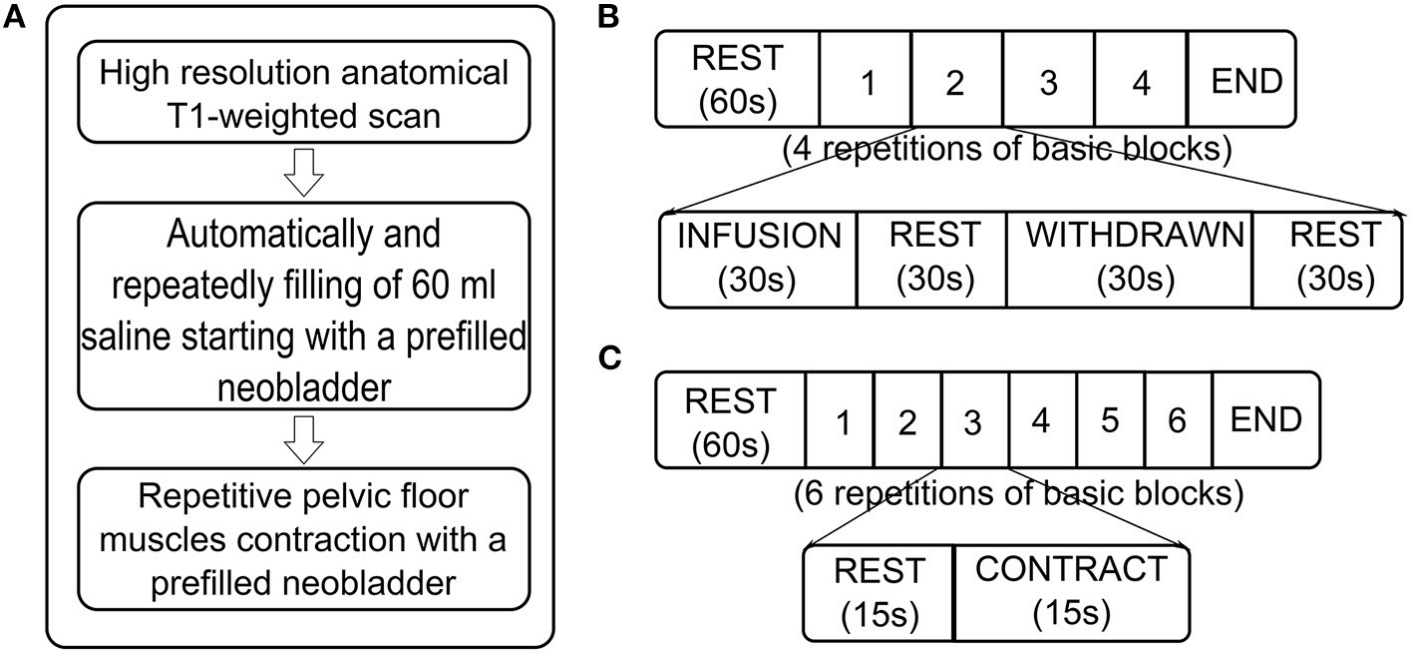
Flow diagram of MRI measurements. **(A)** Sequences of MRI scans. **(B)** Scan paradigm of task-related functional MRI (fMRI) about neobladder volume sensation. Starting with a “baseline” rest (60 s, no specific stimulus or task was performed), it consisted of four repetitive blocks, each with four conditions: (1) automated infusion of 60 ml of body warm saline into the neobladder; (2) rest; (3) withdrawal of 60 ml of saline from the neobladder; and (4) rest. **(C)** Scan paradigm of task-related fMRI about continence. It started with a “baseline” rest (60 s, no specific stimulus or task is performed) and consisted of six repetitive blocks, each with two conditions: (1) rest and (2) pelvic floor muscle contraction.

High-resolution structural T1-weighted images (T1: turbo field echo; TR = 8.2 ms; TE = 3.7 ms; matrix size 198 × 192; 160 sagittal slices; voxel size = 1 mm × 1 mm × 1 mm) were acquired coplanar with functional scans (8, 9) (Figure 1A).

#### Task-Related fMRI: Neobladder Volume Sensation

We recorded a structural brain image followed by repeated blocks of functional brain scans. The neobladder was filled *via* an MR injection system (Spectris Solaris EP, MEDRAD, USA) at 2 ml/s until the patient reported a strong desire to void or to maximal bladder capacity (according to urodynamics before fMRI scan); if the subject had no obvious bladder sensation or urodynamics report, at most 400 ml were used. After the patient rested for 60 s, 60 ml of saline solution were repeatedly infused into and withdrawn from the bladder at 2 ml/s, with 30 s of rest between infusion and withdrawal. This process was repeated for four cycles (10–13). These processes were intended to obtain bladder volume sensory-related brain activation signals by stimulating different bladder volumes (Figures 1A,B).

#### Task-Related fMRI: Continence

A block design was used in this section. The neobladder was emptied and then filled *via* the MR injection system at 2 ml/s until the patient reported a strong desire to void or maximal bladder capacity was reached (according to urodynamics before fMRI scan). If no obvious bladder sensation or urodynamics were reported, the volume was at most 400 ml. After the patient rested for 60 s, the patient was instructed to contract the pelvic floor muscles for 15 s and repeat the above process for six cycles (13–15). These processes were intended to mimic the inhibition of micturition reflex triggering since the subjects with a full neobladder were asked not to urinate (Figures 1A,C).

#### fMRI Scanning

Two functional scans, one lasting 9 min and another lasting 3 min, were acquired (echo-planar T2^*^-weighted gradient-echo; TR = 3,000 ms; TE = 35 ms; flip angle = 90°; matrix size 128 × 128; 33 axial slices; 1.8-mm in-plane resolution; and 4 mm thick) spanning the dorsal-ventral extent of the anterior cingulate cortex.

#### Data Postprocessing

The task-related fMRI images were preprocessed with Data Processing Assistant for Resting-State fMRI (DPARSF, http://rfmri.org/DPARSF) based on Statistical Parametric Mapping (SPM12, http://www.fil.ion.ucl.ac.uk/spm). First, we converted the image format from DICOM to NIFTI. Then, after the removal of the first 10 volumes of fMRI, the remaining volumes were corrected for different signal acquisition times. The functional volumes were motion corrected using a six-parameter rigid-body transformation. The aligned images were coregistered to T1 anatomical images and then subsequently normalized to Montreal Neurological Institute (MNI) space (3 mm^3^ isotropic) using the Diffeomorphic Anatomical Registration using Exponentiated Lie algebra (DARTEL) tool (16). Each normalized scan was smoothed with a Gaussian kernel full width at half maximum (6 × 6 × 6 mm) to reduce residual noise and inhomogeneity between individual brain images.

Given a possible confounding effect of micromovements on intrinsic functional connectivity, we calculated the framewise displacement (FD) values for each subject using the Jenkinson formula, which reflects the temporal derivative of the movement parameters (17, 18).

Each subject was analyzed separately [first-level analysis] using a general linear model convolved with a canonical hemodynamic response function (19). The design matrix of the general linear model in the neobladder volume sensation task for the first-level analysis consisted of the following three conditions: INFUSION, WITHDRAWAL, and REST (30 s). The design matrix of the general linear model in the neobladder continence task for the first-level analysis consisted of the following two conditions: CONTRACTION and REST (15 s). The first-level contrasts were defined by subtraction logic (e.g., [1–1 0 0] to calculate the difference between INFUSION and WITHDRAW). Individual statistical maps were evaluated with a threshold of *P* ≤ 0.001 (uncorrected). The resulting statistical maps were included in group statistics [second-level analysis] using a one-sample *t*-test. The results were considered significant if they had a voxel *P* ≤ 0.001 and a cluster *P* ≤ 0.05 following Gaussian random field (GRF) correction.

## Results

Sixteen patients met the criteria and provided written informed consent, three of whom failed catheter insertion due to a lower urinary tract. A total of 13 subjects were scanned. The average age was 62.10 ± 9.48 years old, and the average postoperative period to the test date was 20.20 ± 13.53 months. The daytime urine continent rate was 100%, and all were able to void voluntarily. The average international prostatic symptom scores (20) during the storage and urination periods were 2.6 and 4, respectively, and the incontinence quality of life score (21) was 72.5 ± 8.13, indicating that all patients had relatively good neobladder function and good quality of life (Table 1 and Supplementary Figure 1).

**Table 1:** Baseline characteristics of subjects.

**Subject ID**	**Age (year)**	**Time after operation (month)**	**Other chronic diseases**	**Daytime continence**	**Nighttime continence**	**I-PSS scored-storage**	**I-PSS scored-urination**	**I-QOL score**	**T stage**	**N stage**	**M stage**
1	72	10	/	+	–	4	5	74	2a	0	0
2	53	17	/	+	+	3	4	75	3b	0	0
3	77	14	Diabetes	+	+	2	4	75	2b	0	0
4	65	35	/	+	+	2	4	78	2a	0	0
5	55	24	Hypertension	+	+	1	4	80	2	1	0
6	62	12	Diabetes	+	–	3	4	58	3a	0	0
7	51	7	/	+	–	3	6	57	1	0	0
8	70	4	/	+	–	3	3	75	a	0	0
9	66	37	/	+	+	3	3	75	3a	0	0
10	50	42	/	+	–	2	3	78	3a	0	0
Mean (SD)	62.10 (9.48)	20.20 (13.53)				2.6 (0.84)	4 (0.94)	72.50 (8.13)			

*I-PSS, international prostatic symptom score; I-QOL, incontinence quality of life score; +, achieve daytime or nighttime continence; –, daytime or nighttime incontinence*.

**Figure 2:**
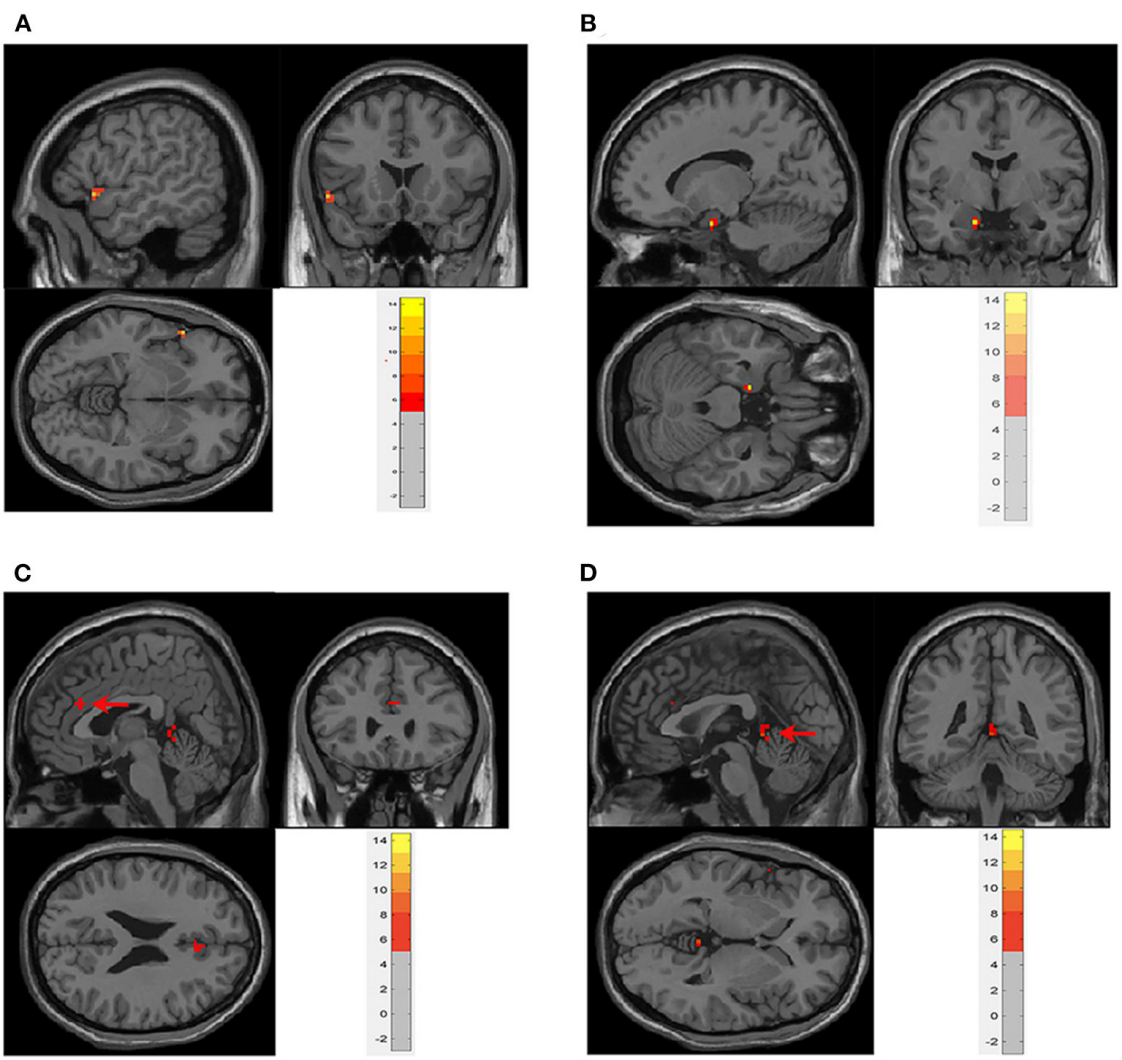
Main effect analysis of neobladder volume sensation in all subjects. There was significant activation in **(A)**. Frontal lobe (−54, 18, and −6); **(B)** parahippocampus (−15, −3, and −24); **(C)** anterior cingulate cortex (ACC) (3, 27, and 24); and **(D)** vermis (0, −42, and 0) (cluster-level *P* ≤ 0.05, GRF correction). The first-level contrasts were defined by subtraction logic between INFUSION and WITHDRAW. GRF, Gaussian random field.

In the neobladder volume sensation task, four cases were excluded because of image artifacts, and a total of nine cases were included in the analysis. The first-level contrasts were defined by subtraction logic to calculating the difference between INFUSION and WITHDRAW. The main effect analysis showed that, when the volume of the neobladder increased, hippocampal gyrus (peak coordinates −15, −3, and −24), frontal lobe (peak coordinates −54, 18, and −6), vermis (peak coordinates 0, −42, and 0), and anterior cingulate gyrus (peak coordinates 3, 27, and 24) activity increased (Table 2 and Figures 2, **4B**) (GRF correction, voxel *P* < 0.001, cluster *P* < 0.05). There were no significant activation changes when calculate the difference between INFUSION and REST or between WITHDRAW and REST.

**Table 2:** Regions responding to neobladder volume sensation and neobladder voluntary continence.

**Task**	**Region**	**Peak MNI coordinate** **(*x*, *y*, and *z*)**	**Peak** ***T*-value**	**Number of voxels**
Volume sensation	Para hippocampal	−15, −3, −24	14.5829	7
	Frontal lobe	−54, 18, −6	11.5966	10
	Vermis	0, −42, 0	6.6498	10
	Anterior cingulate cortex	3, 27, 24	6.5075	9
Continence	Thalamus, caudate nucleus	12, −12, 21	−15.7218	6

*The first-level contrasts were defined by subtraction logic between INFUSION and WITHDRAW. MNI is the human brain coordinates of the Montreal Institute of Neurology. GRF correction, voxel P < 0.001, cluster P < 0.05*.

**Figure 3:**
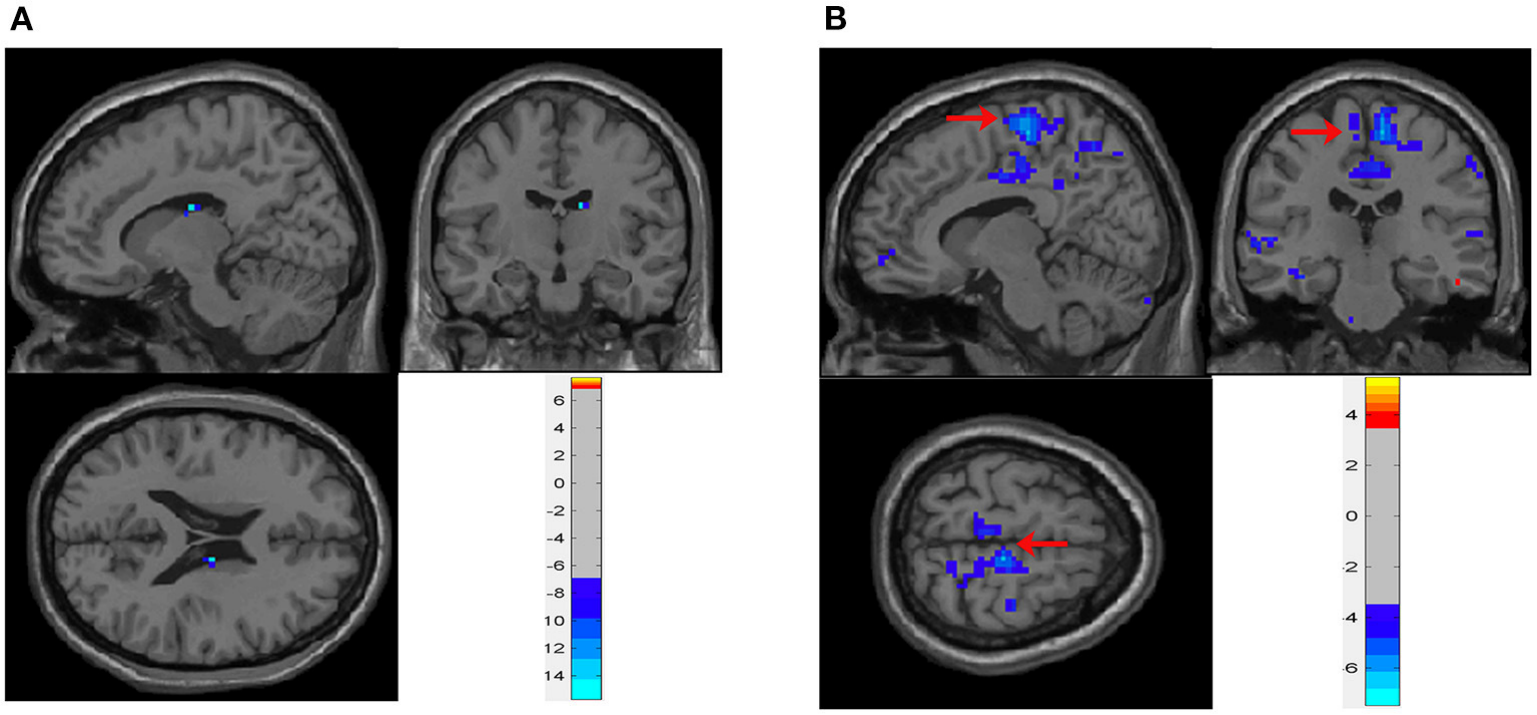
Brain activity during neobladder continence. **(A)** Main effect analysis of neobladder continence in all subjects. There was a significant activation decrease in the thalamus and caudate nucleus (12, −12, and 21) (cluster-level *P* ≤ 0.05, GRF correction). **(B)** Single-subject analysis of neobladder continence in subject no. 10. SMA showed a significant decrease in activation (arrows, also shown in Table 3) (cluster-level *P* ≤ 0.05, GRF correction). The first-level contrasts were defined by subtraction logic between CONTRACTION and REST. GRF, Gaussian random field; SMA, supplementary motor area.

**Figure 4:**
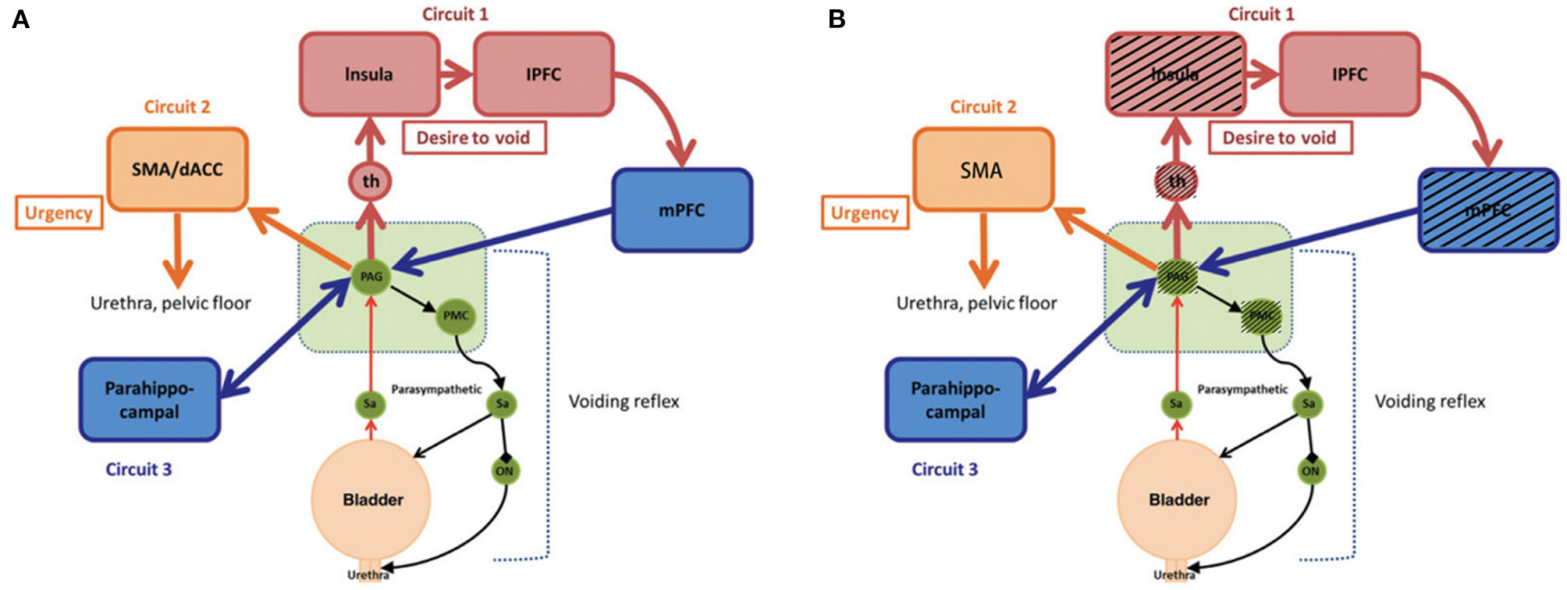
Simple working model of the lower urinary tract control system. **(A)** Reproduced from De Groat et al. (22). Neural control of the lower urinary tract in healthy people. **(B)** Modified from **(A)**. Black shading shows regions active in healthy bladders but not in neobladders, which is a hypothesized difference of mechanism based on small sample size. PAG, periaqueductal gray; PMC, pontine micturition center; th, thalamus; mPFC, medial prefrontal cortex; lPFC, lateral prefrontal cortex; SMA, supplementary motor area; dACC, dorsal anterior cingulate cortex; Sa, Sacral spinal cord; ON, Onuf's nucleus.

In the neobladder continence task, five subjects who had FD > 0.3 mm or translation > 3 mm or rotation > 3° were excluded. And due to the severe image artifacts, two subjects were excluded. A total of six cases were included in the analysis. The main effect analysis showed that, when patients repeatedly contracted the pelvic floor muscles to simulate urine control, the thalamus and caudate nucleus (peak coordinates 12, −12, and 21) were activity decreased (Table 2 and Figures 3A, 4B) (GRF correction, voxel *P* < 0.001, cluster *P* < 0.05). In addition, activation changes in the supplementary motor area (SMA) were observed in all six subjects in the single-subject analysis but not in the overall group analysis, of whom five had bilateral activation, and one patient had only left-side changes. Five patients had decreased activation, and one had increased activation (Table 3 and Figure 3B). Decreased activation of the bilateral frontal orbital cortex was observed in all six subjects in the single-subject analysis but not in the overall group analysis (Table 3).

**Table 3:** Regions responding to neobladder voluntary continence in single-subject analysis.

**Subject**	**Bladder volume^**a**^ (ml)**	**Activated regions**			
		**SMA**		**Frontal orbital cortex**	
		**Left**	**Right**	**Left**	**Right**
4	250	–	–	–	–
5	240	–	–	–	–
6	300	/	–	–	–
7	300	+	+	–	–
9	250	–	–	–	–
10	300	–	–	–	–

*The first-level contrasts were defined by subtraction logic between CONTRACTION and REST. SMA, Supplementary motor area. ^a^In this paradigm, neobladder was prefilled with saline until the patient report a strong desire to void or to maximal bladder capacity (according to urodynamics before fMRI scan) if no obvious bladder sensation and urodynamics report, at most 400 ml. +, Activation increase; –, activation decrease; /, no activation change*.

## Discussion

The physiological functions of the lower urinary tract are to store and empty in appropriate times and conditions. Involuntary micturition is largely intact at birth, while the inhibition of micturition is achieved during a developmental hierarchy of sensory–motor learning; reinforcement and is not a “built-in” behavior present at birth, and the supraspinal control of continence is more complicated (5). Determining the supraspinal control of the lower urinary tract is of great significance for the treatment of lower urinary tract functional diseases.

The exploration of supraspinal control of lower urinary tract functional function has a long history. Before the development of functional imaging technology, scientists evaluated brain activity through animal experiments or observation of the clinical symptoms of patients with brain injury. Over the past 15 years, functional brain imaging has emerged as the most powerful technique for studying human brain function, particularly for understanding the relationship between activity in certain brain areas and specific functions (6). Since 1996, the Japanese scientist Fukuyama and his coworkers have used functional imaging to reveal the functional activities of the brain during urination in healthy men for the first time (23). More brain regions related to micturition function are being elucidated with the development of functional brain imaging. De Groat et al. (22) suggested that control was exerted by three “loops” based on the results of existing research: Circuit 1: PAG-thalamus-insula-prefrontal cortex-PAG, which is mainly responsible for urination decisions; Circuit 2: PAG-dorsal anterior cingulate cortex/SMA-sphincter/pelvic floor muscles, related to continence; and Circuit 3: parahippocampal PAG, a hippocampal loop, which is active when the bladder fills slowly without causing a strong bladder sensation (5, 22) (Figure 4A). In addition, some studies have revealed changes in brain activity in disease conditions, such as urinary retention, urinary incontinence (13), overactive bladder (10), spinal cord injury (24), and Parkinson's disease (25), explaining the disease occurrence from the perspective of supraspinal regulation and providing new possibilities for treatment. BCa has high morbidity worldwide (1). OIN is the desirable urinary diversion option after RC (2). Reducing postoperative lower urinary tract dysfunction and enabling patients to obtain a greater quality of life have always been important topics. The lower urinary tract neuromodulation of OIN has undergone greater changes after surgery than with the normal bladder. Coupled with postoperative rehabilitation training, the acquisition of new bladder sensation and control causes greater changes in related neural regulation. However, there has no related research for reference. In this study, 18 male patients with BCa undergoing laparoscopic RC and OIN were investigated using task-related fMRI technology.

### Neobladder Volume Sensation

In this study, saline solution was repeatedly infused into and withdrawn from the neobladder, and functional magnetic resonance scanning was performed at the same time to obtain brain region activation while the bladder volume changed (10–13). In this experiment, the regions activated during bladder filling included the parahippocampus (peak coordinates −15, −3, and −24), frontal lobe (peak coordinates −54, 18, and −6), vermis (peak coordinates 0, −42, and 0), and anterior cingulate cortex (peak coordinates 3, 27, and 24) (Table 2 and Figure 2).

In the original bladder, hippocampal activation occurs in the small bladder volume and when the bladder volume slowly increases (26). At this time, the volume change does not sufficiently stimulate the volume receptors on the bladder wall, and subjects usually have no obvious bladder sensation; therefore, brain regions that are active in high volumes are not active in this condition, and the parahippocampal-PAG circuit is activated. Among women with idiopathic inability bladder sensation, restoration of bladder sensation by sacral neuromodulation leads to changes in a similar brainstem/parahippocampal network (27), suggesting that this network could be the route by which the PAG normally monitors bladder behavior and exchanges bladder-related signals with the rest of the brain (5). In this study, the neobladder was filled rapidly until urgency or the maximum bladder volume was reached. No activation was observed in the insula, thalamus, and PAG, which are related to high bladder volumes. According to recent studies, it might be suggested that the neobladder cannot completely establish the original bladder volume sensory response, while the hippocampal alternative plays a role. The anterior cingulate belongs to the limbic system and is responsible for motivation and modulation of bodily arousal states. It is also related to cardiac rhythm and could regulate visceral activity through beta- and alpha-adrenergic receptors on these peripheral organs (28, 29). Anterior cingulate gyrus activation not only increases during bladder filling in the original bladder but is also involved in intestinal sensation (30–33). We believe that the activation of the anterior cingulate gyrus in the neobladder is a reflection of the intestinal sensation of the intestinal segment used for the neobladder structure. The prefrontal cortex is activated in the original bladder in high bladder volumes and is generally considered to be involved in micturition decisions (34, 35). The cerebellum has traditionally been considered a center of secondary motor control and coordination. A growing body of research has shown that the cerebellum influences visceral functions. studies by Nishizawa et al. (36) and Blok et al. (37) suggested that the cerebellum plays a role in both the collecting and emptying phases. Siffert et al. (38) found that children with postoperative cerebellar mutism suffered from long-lasting urinary and fecal incontinence. These studies indicate that the cerebellum plays a role in micturition, but its specific role remains unclear (5).

Based on the results of this study, we considered that the original volume sensation was absent in OIN and that the original intestinal visceral volume sensation was preserved. The PAG and PMC activation is absent in OIN.

### Continence

Subjects were asked to repeatedly contract their pelvic floor muscles during full bladder to mimic the inhibition of micturition reflex triggering since the subjects were asked not to urinate. The caudate and thalamus were active in this task. The caudate belongs to the basal ganglia. The basal ganglia, such as the putamen, play a role in lower urinary tract activity (14, 15, 39), and in visceral function, the signal between the pons and limbic system may be relayed by the basal ganglia (40, 41). Seseke et al. (15) observed changes in the fMRI response in patients before and after radical prostatectomy with special emphasis on pelvic floor muscle control, and the basal ganglia and thalamus were activated before and after surgery. Supplementary motor area activation has been observed during repeated contractions of the pelvic floor muscles in many studies (14, 15, 42). In this study, activation of the supplementary motor area was observed in five of the six subjects in the single-subject analysis but not in the overall group analysis. Failure to detect activation in the group analysis might have been due to the relatively longer postoperative time interval, and subjects in our study accepted different rehabilitation training, leading to different pelvic floor muscle activity patterns. Similarly, in the frontal orbital frontal cortex, which is related to micturition decisions, activity was observed in all six subjects in the single-subject analysis but not in the overall group analysis.

### Limitations

The main limitation of this study was the small sample size, which may affect the reliability of the results. Another limitation was that this study fails to evaluate brain activity during micturition because voiding while supine may pose a challenge to individuals who can void normally while standing. The patients with OIN are void by relaxing the pelvic floor and, if necessary, by abdominal straining (Valsalva maneuver), which may lead to the difference in the supraspinal program.

### Conclusion

A complex supraspinal program is involved during OIN control and is significantly different from that of the normal bladder. We found that the original bladder volume sensation was absent in OIN and that the original intestinal visceral volume sensation was preserved. In addition, the PAG and PMC activation is absent in OIN. In voluntary urinary control, we showed that activation of the micturition circuit is different from that with the original bladder. These differences could be attributed to surgically induced damage, and different rehabilitation training could play a role.

## Data Availability Statement

The raw data supporting the conclusions of this article will be made available by the authors, without undue reservation.

## Ethics Statement

The studies involving human participants were reviewed and approved by Research Ethics Committee, The Sun Yat-sen Memorial Hospital, Sun Yat-sen University. The patients/participants provided their written informed consent to participate in this study.

## Author Contributions

All authors listed have made a substantial, direct, and intellectual contribution to the work and approved it for publication.

## Funding

This work was supported by the National Natural Science Foundation of China (Nos: 81672550, 81974395, 81772733, and 81972384) to ZG, the Guangdong Basic and Applied Basic Research Foundation (No: 2019A1515011437), the Guangzhou Science and Technology Cooperation Program (Foreign research and development cooperation) (No: 201807010087), the Sun Yat-sen University Clinical Research 5010 Program (No: 2019005), the Sun Yat-sen Clinical Research Cultivating Program (No: 201702), the Guangdong Province Key Laboratory of Malignant Tumor Epigenetics and Gene Regulation (No: 2020B1212060018OF006), the Guangdong Science and Technology Department (2017B030314026), Guangdong Scientific Research Projects (Nos: 2016A020215011 and 2021A1515010223) to ZG, and was funded by the Chinese national scholarship.

## Conflict of Interest

The authors declare that the research was conducted in the absence of any commercial or financial relationships that could be construed as a potential conflict of interest.

## Publisher's Note

All claims expressed in this article are solely those of the authors and do not necessarily represent those of their affiliated organizations, or those of the publisher, the editors and the reviewers. Any product that may be evaluated in this article, or claim that may be made by its manufacturer, is not guaranteed or endorsed by the publisher.

## Supplementary Material

Supplementary Figure 1 Urodynamic study at 6 months after reconstruction of the ileal orthotopic neobladder. **(A)** Uroflowmetry; **(B)** pressure-flow study. Flow, urinary flow rate; Pves, total bladder pressure; Pabd, abdominal pressure; Pdet, detrusor (neobladder) pressure.
