# Supplementary figures and images for: Neuroimaging Study Investigating the Supraspinal Control of Lower Urinary Tract Function in Man With Orthotopic Ileal Neobladder
Source: Front Surg. 2021 Dec 7;8:751236. doi: 10.3389/fsurg.2021.751236 (PMC8688399; doi:10.3389/fsurg.2021.751236)

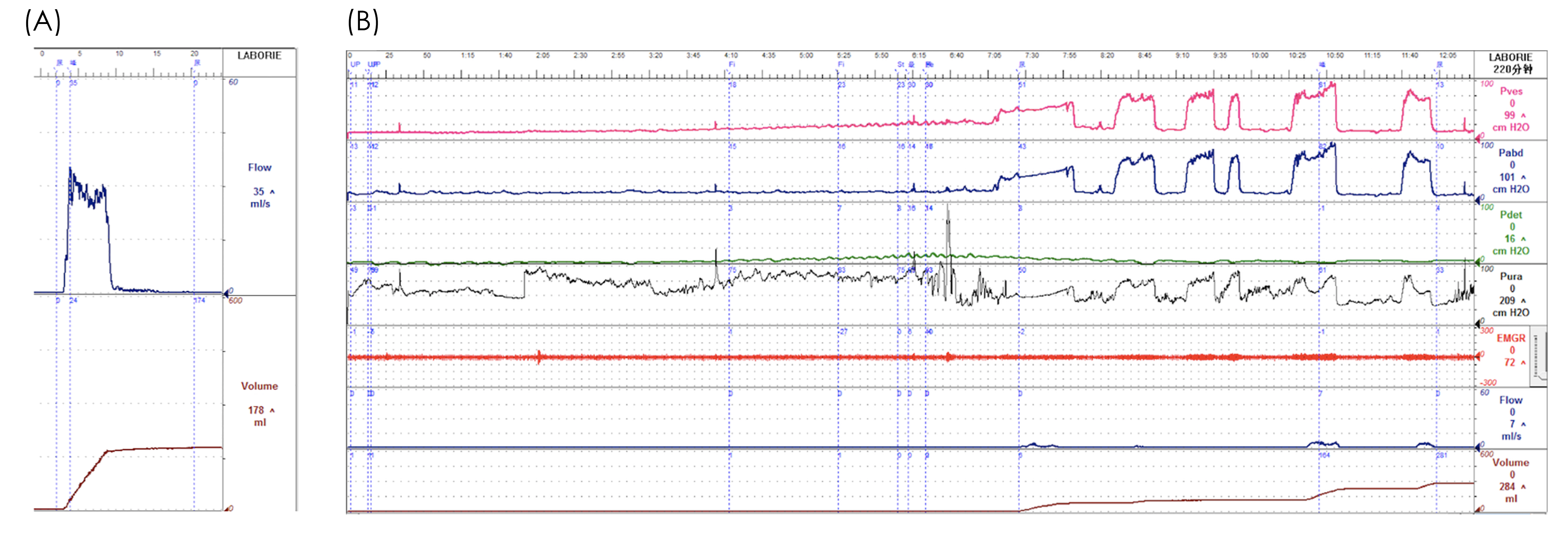

Supplement: Supplementary Figure 1 — Urodynamic study at 6 months after reconstruction of the ileal orthotopic neobladder. (A) Uroflowmetry; (B) pressure-flow study. Flow, urinary flow rate; Pves, total bladder pressure; Pabd, abdominal pressure; Pdet, detrusor (neobladder) pressure. [file Image_1.TIF]
